# *In vitro* and *in silico* study on the seeds of *Veitchia merrillii* on trematode worms

**DOI:** 10.14202/vetworld.2024.1336-1347

**Published:** 2024-06-21

**Authors:** Farida Athaillah, Muhammad Hambal, Heni Vanda, Frengki Frengki, Wahyu Eka Sari

**Affiliations:** 1 Department of Parasitology, Faculty of Medicine Veterinary, Syiah Kuala University, Banda Aceh, Indonesia; 2 Department of Pharmacology, Faculty of Medicine Veterinary, Syiah Kuala University, Banda Aceh, Indonesia; 3 Department of Biochemistry, Faculty of Medicine Veterinary, Syiah Kuala University, Banda Aceh, Indonesia

**Keywords:** Molecular docking, QSAR, Trematoda, *Veitchia merillii*

## Abstract

**Background and Aim:**

The potential of plants as anthelmintics is very large, but there is still very little research conducted in the search for effective, safe, easily obtained, and affordable anthelmintic candidates. Palem putri (*Veitchia merrillii*) is an ornamental plant that is interesting to study because it is included in the areca nut group which is reported to have strong abilities as anthelmintics. The study aims to evaluate the anthelmintic efficacy of *Veitchia merrillii* against trematode worms such as *Paramphistomum* spp. and *Fasciola hepatica*.

**Materials and Methods:**

This research employs both *in vitro* and computational techniques. An anthelmintic *in vitro* test was carried out on *Paramphistomum* spp. worms at concentrations of 10%, 25%, and 40% (gr/v), assessing mortality index as the observable outcome, followed by a histopathological investigation of the deceased worms for tissue and cellular damage evaluation. Seventeen compounds from *V. merrillii* seeds were studied *in silico* for their anthelmintic activity against *F. hepatica* worms using the quantitative structure-activity relationship technique, molecular docking, and Lipinski’s rule analysis for orally administered medication.

**Results:**

About 25% and 40% extracts of *V. merrillii* damaged the tegument organs in the worms. Seventeen compounds in *V. merrillii* seed extract, on average, yielded a higher anthelmintic index on *F. hepatica* than praziquantel. Eleven of the 17 compounds exhibit stronger affinity than praziquantel, with routine and gallic acid being the top two ligands (∆Gbinding values: −11.65 kcal/mol and −11.07 kcal/mol, respectively). According to Lipinski’s rule analysis, only routine compounds cannot be orally administered.

**Conclusion:**

The seeds of *V. merrilli* have potential as an anthelmintic agent for *Paramphistomum* spp. at concentrations of 25%–40% (gr/v).

## Introduction

Parasitic diseases significantly impact global health and livestock productivity. Small ruminants are susceptible to gastrointestinal infections from nematodes, trematodes, and cestodes [1]. In Indonesia, the presence of *Haemonchus* spp., *Fasciola gigantica*, and *Paramphistomum* spp. worms pose a significant threat to young livestock. *Haemonchus placei*, a type of gastrointestinal nematode, can hinder growth and lead to death in both cattle and sheep. This parasite, which feeds on blood, is responsible for numerous deaths, predominantly among the young. In addition, these gastrointestinal worms are easily resistant to commercial anthelmintics, especially in countries that have many small ruminants [2]. *Paramphistomum* spp. worms, including both adults and young images, have been known to cause *Paramphistomiasis*. The infection can progress from the rumen to the abomasum and small intestine. Ruminants are at risk from this disease [3–6]. Livestock is commonly infected by *Paramphistomum cervi*, *Paramphistomum ichikawai*, *Paramphistomum gotoi*, and *Paramphistomum scotiae*. These trematodes, including *Fasciola hepatica* and *F. gigantica* cause infections (fasciolosis) in cattle, sheep, and other ruminants. Fasciolosis causes economic losses amounting to USD 31.65 million annually. Worm infestation in the liver and gallbladder of ruminants can impede growth, decrease production, render the liver unsuitable for consumption, and potentially result in fatality. The necessary measures should be implemented to combat losses from fasciolosis resulting from infections with these two worms.

Using natural anthelmintic sources as an alternative can eliminate worms. The availability and affordability of numerous raw materials, easy financing, and lower chances of resistance make plants attractive alternatives for anthelmintic production. It is estimated that more than 20,000 plant species have been used worldwide to treat various types of diseases, most of them as antibiotics [7–9], some of which also study the anthelmintic properties of these medicinal plants. In Cameroon and Ghana, the medicinal use of *Anogeissus leiocarpus*, *Khaya senegalensis*, *Euphorbia hirta*, *Annona senegalensis* water extracts, and *Parquetina nigrescens* is reported for their anthelmintic properties [10]. Vidyadhar *et al*. [11] revealed the anthelmintic property of *Enicostemma littorale*. Jeyathilakan *et al*. [12] found that *Cymbopogan nargus* and *Azadirachta indica* were useful for treating *F. gigantica* infections. *Nigella sativa* extract and ivermectin were reported to be effective as an anthelmintic by Shalaby and El-Moghazy [13]. Pal and Pandap [14] explained that the anthelmintic potential is also found in the *Cynodon dactylon* plant, which is traditionally used as a cure for epilepsy, diarrhea, dysentery, cancer, coughs, wounds, hypertension, and rheumatism by people in India. *Gynandropsis gynandra* and *Buchholzia coriaceae*’s leaf and root extracts are active against *F. gigantica*, *Tenia solium*, and *Pheritima* pashuma trematode worms [15]. Zahir *et al*. [16] explained that ethyl acetate extract of *Achyranthes aspera* leaves, acetone and chloroform extract of *Anisomeles malabarica* leaves, methanol extract of *Gloriosa superba* flowers, and methanol extract of *Ricinus communi*s leaves have the potential to be used in controlling parasites *Paramphistomum cervi*, *Rhipicephalus* (Boophilus) *microplus*, *Anopheles subpictus*, and *Culex tritaeniorhynchus*. Ethanol extract of *Jatropha curcas* seeds inhibits *Haemoncus contortus* nematodes [17], while *Allium sativum* and *Lawsonia inermis* extracts exhibit fluidal activity against *F. gigantica* [18]. In Indonesia, most people have utilized areca nuts as an anthelmintic medicine [19–29]. The *Veitchia merrillii*, also referred as its areca nut family name *Arecaceae* [23,30,31], is a palm species. In-home gardens, offices, parks, and urban roadsides, this plant is popularly used as an ornamental plant. Beyond its economic significance, this plant is also found to have fatty acids, including palmitic acid, oleic acid, and linoleic acid [32, 33]. Using high-performance liquid chromatography (HPLC), Vafaei [34] identified gallic acid, caffeic acid, vanillic acid, syringic acid, pyrogallol, quinic acid, and naringenin as active compounds. The last two compounds are the most frequently occurring components in the fruit of this plant. Hamzah *et al*. [23] have reported that this plant is effective in killing *Ascaridia galli* worms *in vitro*. This article provides information on *V. merrillii*’s anthelmintic properties against *F. hepatica* and *Paramphistomum* spp. trematode worms.

This study aimed to investigate the antitrematode properties of *V. merrilli* fruit seeds using both *in vitro* and *in silico* techniques. The *in vitro* mortality and histology of *Paramphistomum* spp. worm tegument was examined following treatment with *V. merrillii* ethanol extract at concentrations of 10%, 25%, and 40%. *In vitro* studies employing QSAR and molecular docking techniques were performed to evaluate the anthelmintic potential of 17 secondary metabolites in *V. merrillii* seeds against *F. hepatica* worms [32–34].

## Materials and Methods

### Ethical approval

This study was conducted *in vitro* using worm parasites and *in silico*, so ethical approval is not required.

### Study period and location

The study was conducted from February to July 2023 in the Parasitology and Pharmacology Laboratory, Faculty of Veterinary Medicine, Syiah Kuala University.

### Materials

The sample consisted of *Veitchia merillii* fruit seeds sourced from the “Blang Padang” park region of Banda Aceh, Indonesia. It was identified by Devi Syafrianti in the Biology Laboratory of Syiah Kuala University. Ethanol 70% was used to extract dried *V. merrillii* fruit seeds for *in vitro* testing on worm motility and mortality. Seventeen *V. merrillii* fruit seed active compounds, downloaded from PubChem and converted in pdb format, were used as ligands against thioredoxin enzyme receptor (pdb id. 2VIM) in an *in silico* test whose hardware specifications was a set of computers with processorCore™ i5-3230M2 Cores chip, 4 Threads@2.6GHz, 4.00 GB DDR3 1600 MHz random access memory, 2GB DDR3 Radeon HD 8670M video graphics array, supported by internet access. While software used was the Molecular Operating Environment (MOE) (V.9 2010, Chemical Computing Group, Inc., Canada) relies on various web servers including http://www.way2drug.com/PASSOnline/predict.php, http://stitch.embl.de, and https://biosig.lab.uq.edu.au/pkcsm. for support.

### Methods

#### Ethanol extraction of V. merrillii fruits seeds

Following the method of Jiraungkoorskul *et al*. [35] with modifications (maceration container connected to automatic stirring tool), we weighed *V. merrillii* seeds to ±5 kg, dried them in the absence of sunlight, and ground them into powder using a blender. The filtrate was taken 3 times after macerating the powder with ethanol solution. In the Rotavapor® R-300 (China), a vacuum rotary evaporator, the macerate was evaporated to yield a thick, ethanol-free extract.

#### Worm motility and mortality test using V. merrillii extract

*Paramphistomum* spp. was collected from the abomasums of cattle slaughtered at the Lambaro Aceh Besar. Worms were transferred to RPMI 1640 (Sigma-Aldrich^®^, USA) medium for motility and mortality testing. Ten worms for each treatment were placed in separate Petri dishes, and 10% (P1), 25% (P2), and 40% (P3) of *V. merrillii* extract were added to each, respectively, in triplicate. Praziquantel served as the positive control (C0), while phosphate-buffered saline (PBS) acted as the negative control (C1). Every 15 min, an index score was used to determine worm motility. The worm’s body movement is assessed. A body moving completely was scored 3, partially moved, 2; alive but not moving, 1; and dead, 0. We confirmed worm death by touching them with a stir bar. A moving worm confirms its life. The worm’s death is confirmed if it remains silent.

#### Histopathological examination

Histopathological preparations were made from each treatment group using dead worms. The examination involves an initial rinse of worm samples with PBS, followed by fixation using 10% buffer neutral formalin, then a stopping point with 70% alcohol, and subsequent dehydration using graded alcohol concentrations (70%, 80%, 90%, and absolute). The tissue in Silol I, II, and III fluids is cleared, infiltrated with liquid paraffin, and then embedded in paraffin to form a paraffin block. 5-μm thick tissue slices were prepared using a microtome, stained with hematoxylin-eosin, and mounted on slides with Entellan® (Merck, Germany) adhesive. Observations were made and recorded as photomicrographs using the Olympus CX31 (Japan) microscope.

#### Literature search for secondary metabolites of V. merrillii plant seeds and download of the smile structure of these compounds through PubChem

Seventeen compounds, including gallic acid, caffeic acid, vanillic acid, syringic acid, pyrogallol, rutin, naringenin, limonene, cis-β-ocimene, allo-ocimene, linalool oxide, linalool, methyl salicylate, eucalyptol, palmitic acid, oleic acid, and linoleic acid, have been isolated from the *V. merrillii* seeds according to Rodríguez-Leyes *et al*. [32] and Vafaei [34] (Table-1).

**Table-1:** Seventeen compounds of secondary metabolites of *Veitchia merrillii* fruit seeds [32, 34].

S. No.	Compounds	Canonical SMILES
1	Gallic acid	C1=C (C=C (C(=C1O) O) O) C(=O) O
2	Pyrogallol	C1=CC(=C (C(=C1) O) O) O
3	Caffeic acid	C1=CC(=C (C=C1C=CC(=O) O) O) O
4	Vanillic acid	COC1=C (C=CC(=C1) C(=O) O) O
5	Syringic acid	COC1=CC(=CC(=C1O) OC) C(=O) O
6	Rutin	CC1C (C (C (C (O1) OCC2C (C (C (C (O2) OC3=C (OC4=CC(=CC(=C4C3=O) O) O) C5=CC(=C (C=C5) O) O) O) O) O) O) O) O
7	Naringenin	C1C (OC2=CC(=CC(=C2C1=O) O) O) C3=CC=C (C=C3) O
8	Limonene	CC1=CCC (CC1) C(=C) C
9	cis-β-ocimene	CC(=CCC=C (C) C=C) C
10	Allo-ocimene	CC=C (C) C=CC=C (C) C
11	Linalool oxide	CC(=CCCC (C)(C1CO1) O) C
12	Linalool	CC(=CCCC (C)(C=C) O) C
13	Methyl salicylate	COC(=O) C1=CC=CC=C1O
14	Eucalyptol	CC1(C2CCC (O1)(CC2) C) C
15	Palmitic acid	CCCCCCCCCCCCCCCC(=O) O
16	Oleate acid	CCCCCCCCC=CCCCCCCCC(=O) O
17	Linoleic acid	CCCCCC=CCC=CCCCCCCCC(=O) O
	Praziquantel (Control)	C1CCC (CC1) C(=O) N2CC3C4=CC=CC=C4CCN3C(=O) C2

#### Determination of the potential of 17 metabolites of V. merillii plant seed compounds as anthelmintic agents in F. hepatica worms based on Way2Drug QSAR analysis

The Prediction of Activity Spectra for Substances (PASS) web server, available at http://www.way2drug.com/PASSOnline/predict.php, was used to predict anthelmintic activity against *F. hepatica* using SMILES data for the test compound. The probability of Pa and Pi varies between 0.000 and to1.000. PASS predictions are interpreted within a flexible range, namely: (i) Pa > Pi values are considered to have the possibility of being active; (ii) if Pa > 0.7, the probability of being experimentally active is high; (iii) if Pa >0.5 but <0.7, there is a chance that it will be experimentally active, but the compound may be different from the known active compound; (iv) if Pa <0.5 the chance of finding activity experimentally is low, but the chance of finding new chemical entities is high [36, 37].

The potential as an anthelmintic for trematodes is an average of 17 secondary metabolites of *V. merrilli* fruit seeds based on the anthelmintic parameter score for *F. hepatica* worms shown by the way2drug webserver varying from 0 to 1, which shows the accuracy of the analysis [38]. The anthelmintic activity of certain compounds against *F. hepatica* was used to determine their Pa scores through QSAR analysis. The similarity of a compound’s structure increases its predictive power.

#### Molecular docking

Through molecular docking analysis, the thioredoxin enzyme from the worm *F. hepatica* serves as the target, chosen due to QSAR method findings indicative of its role in the anthelmintic mechanism. Seventeen *V. merrillii* seed compound structures, downloaded from PubChem as “canonical SMILES” and converted to the pdb format, were used as test ligands. The 2VIM pdb structure for thioredoxin enzyme was retrieved from www.rscb.org. The initial molecular docking process involved optimizing ligand and receptor structures through adding hydrogen atoms, partial energy, and adjusting the system energy to a minimum for maximum binding affinity. The MOE application’s site finder is used to trace the binding site of the 2VIM receptor during the docking process. A ”site” matching the docking target reported by Shukla *et al*. [39] was chosen. The selected test ligands are then docked. ∆Gbinding values for docking results are displayed in a table and visualized in the form of 2-dimensional image (MOE LigPlot; Chemical Computing Group, Canada).

#### Sequence alignment and superposition of the 3D structures

Alignment was performed to observe the differences between the sequences that make up the thioredoxin enzyme between the worm species *F. hepatica* and the host *Bos taurus* using the sequence alignment method with the help of the web server https://www.ebi.ac.uk/Tools/msa/clustalo/. Furthermore, the 3D thioredoxin enzyme structures of the two species were superimposed to observe differences in their geometric conformations.

#### Analysis of drug-likeness, absorption, distribution, metabolism, excretion, and toxicity of 17 metabolites of V. merrillii fruit seed compounds

Absorption, distribution, metabolism, and excretion (ADME) analysis evaluates a compound’s potential behavior within the body, encompassing ADME. A compound’s suitability for oral drug administration can be determined by Lipinski’s rule of five, which states that a molecule must have a molecular mass under 500 daltons, a LogP value below five, no more than five hydrogen donor bonds, no more than 10 hydrogen acceptor bonds, and a molar refraction between 40 and 130. ADME and compound toxicity were predicted using the pkCSM (predicting small-molecule pharmacokinetic properties using graph-based signatures) web server, as described by Pires *et al*. [40].

## Results and Discussion

### Anthelmintic treatment

The abomasum samples obtained from the Lambaro Aceh Besar slaughterhouse only found *Paramphistomum* spp. worms, so *in vitro* treatment was only carried out on this type of worm. The results show that 40% ethanol extract of *V. merrillii* fruit seeds has the strongest anthelmintic power compared with concentrations of 10% and 25% against *Paramphistomum* spp. worms. All worms died within 80 min after soaking. These results were even better than those of the positive control. The anthelmintic power parameters observed were the motility and mortality scores of *Paramphistomum* spp. worms after being given ethanol extract of *V. merrillii* fruit seeds at 30, 60, 90, and 100 min after incubation.

The negative control group demonstrated continuous movement for all *Paramphistomum* spp. worms for 30, 60, 90, and 100 min following incubation (score 3). 30 min after incubation, all *Paramphistomum* spp. worms in the control group were still active (scores 3); 60 min after incubation, four worms had entire body movement (scores 3) and six had incomplete body movement (scores 2); 90 min after incubation, four worms were alive with no movement (scores 1) and nine were died (scores 0); 100 min after incubation, one worm had no movement but was still alive (scores 1) and nine were died (scores 0).

Eight *Paramphistomum* spp. worms exhibited active movement (score 3) and two had partial movement (score 2) during 30 min of incubation with 10% ethanol extract from *V. merrillii* fruit seeds. Six tails remained alive with no movement (score 1) while one worm died (score 0) within 60 min post-incubation. All worms (score 0) ceased to move at 90 and 100 min post-incubation.

Seven *Paramphistomum* spp. worms, with some part of their bodies still moving, were found in the group given 25% ethanol extract, while three worms remained alive with part of their bodies active during the 30-min incubation; however, one tail was only alive with part of the worm body moving, seven tails did not move but remained alive, and two tail worms were died after 60 min; unfortunately, all worms had died by 90 and 100 min.

In the group given 40% ethanol extract of *V. merrillii* fruit seeds, three *Paramphistomum* spp. worms were found to still be actively moving throughout their bodies (score 3), and seven worms showed that they were still alive with part of the worm’s body moving (score 2) during the 30 incubation period; six worms did not move but were still alive (score 1), four worms died (score 0) during 60 min post-incubation, and all worms died (score 0) at 90 and 100 min post-incubation. The *in vitro* experiment results are presented in Table-2.

**Table-2:** Motility of *Paramphistomum* spp. worms in *Veitchia merrillii* fruit seeds extract and control.

Time (min)	Score	Treatment (n=10)				

		C0	C1	P1	P2	P3
30	3	10	10	8	7	3
	2			2	3	7
	1					
	0					
60	3	10	4			
	2		6	3	1	
	1			6	7	6
	0			1	2	4
90	3	10				
	2					
	1		4			
	0		6	10	10	10
100	3	10				
	2					
	1		1			
	0		9	10	10	10

*V. merrillii* fruit seeds have been identified as rich sources of alkaloids, phenol-flavonoids, and tannins, according to Balqis *et al*. [19]. Vafaei [34] identified gallic acid, vanillic acid, kaffic acid, syringic acid, nagarin, pyrogallol, and routine flavonoids as the constituents of *V. merrillii* seeds using HPLC analysis. Tannins are polyphenolic compounds with astringent or protein-precipitating properties. Damaging the worm’s protein membrane with this ability leads to paralysis and death. Tannins can hinder the nutritional intake of worms by suppressing their digestive metabolism [41, 42]. Mali and Mehta [43] reported the uncoupling mechanism of oxidative phosphorylation and cuticular glycoprotein binding in *A. galli* worms. This mechanism may occur in *F. hepatica*. The root extract of *Adhatoda vasica* plant inhibits nerve impulses in *A. galli* worms, leading to paralysis [44]. Alkaloids enhance gastrointestinal contractility, amplifying peristaltic waves to expel parasites from the digestive system [44]. *V. merrillii* seeds’ flavonoids inhibit the development of worms and filarial parasites [10]. Lakshmi *et al*. [45] reported antifilarial activity of naringenin, flavone, hesperetin, rutin, naringenin, and chrysin against *Brugia malayiin*. Against various parasites, triterpenoids demonstrate anthelmintic properties. According to Mali and Mehta [43], extracts from *Mimusops elengi* Linn contain triterpenoids and saponins which lead to paralysis and death of worms. Strychnos spinosa leaves’ triterpenoids were reported by Hoet *et al*. [46] to inhibit *Trypanosoma brucei*’s development *in vitro*. Previously, the insecticidal bioactivity of *A. indica* leaf triterpenoids against *Aedes aegypti* mosquito larvae was proven by Siddiqui *et al*. [47].

**Figure-1:**
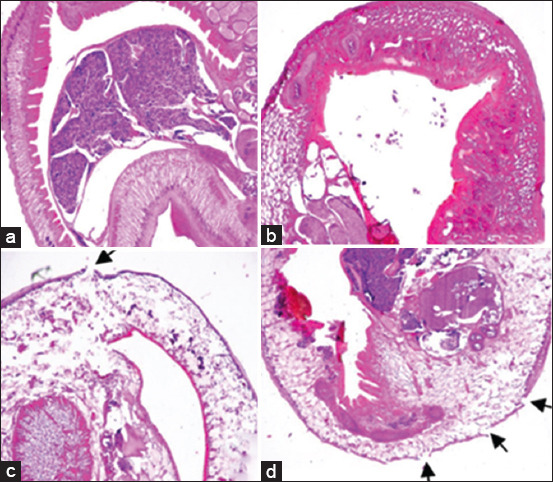
Tegument of *Paramphistomum* spp. (a) Negative control. (b) 10% *V. merrillii* extract. (c) 25% *V. merrillii* extract. (d) 40% *V. merrillii* extract. The arrows indicate a damaged tegument. *V. merrillii*=*Veitchia merrillii*.

Histopathological observations revealed ongoing mortality effects. The intention was to examine organ damage in *Paramphistomum* spp. worms caused by *V. merrillii* fruit seed extract administration. 25% and 40% *V. merrillii* seed extract-induced tegument damage in *Paramphistomum* spp., as evidenced by their thinned and disintegrated layers (Figure-1). The tegument layer significantly contributes with crucial enzymes such as acid and alkaline phosphatases, amino peptidase, glutathione S-transferase, acetylcholine esterase, glucose transporter, serine hydrolase, and glycolytic enzymes [48]. The tegument layer, as a sensory organ, adapts to the environment by absorbing exogenous food ingredients [49].

The PASS prediction web server was used to predict the biological activity of each test compound. The main biological activity of 17 *V. merrillii* fruit seed compounds is enhanced by various mechanisms as revealed in Table-3. The Pa > Pi value in this study indicated the biological activity of all test compounds. The average anthelmintic Pa value, determined by anthelmintic activity, among the 17 test compounds was 0.340 (±0.123), falling within a range of 0.145–0.580. The linoleic acid compound had the greatest Pa value, while limonene had the least17 *V. merrillii* seed compounds exhibit stronger anthelmintic potential than praziquantel, as determined by the QSAR method.

**Table-3:** Prediction scores for 17 *Veitchia merrillii* fruit seed compounds which are associated with anthelmintic effects on *Fasciola hepatica* worms.

S. No.	Compounds	Anthelmintic	Microtubule formation inhibitor	Ca^+2^ channel activator	Thioredoxin inhibitor	Cholinergic antagonist	Agonist apoptosis	Ubiquinol- cytochrome- c reductase inhibitor	TP53 expression enhancer	Neurotransmitter antagonist	Caspase 3 stimulant
Praziquantel (Control)		0.305	0	0.125	0	0.284	0	0	0	0.417	0
1	Gallic acid	0.356	0.234	0.612	0.409	0.573	0.562	0.915	0.718	0.64	0.413
2	Pyrogallol	0.524	0.23	0.69	0.864	0.641	0.775	0.914	0.744	0.66	0.507
3	Caffeic acid	0.277	0.285	0.521	0.563	0.482	0.711	0.843	0.776	0.523	0.579
4	Vanillic acid	0.253	0.309	0.363	0.488	0.57	0.512	0.922	0.713	0.556	0.64
5	Syringic acid	0.271	0.276	0.359	0.363	0.566	0.538	0.925	0.712	0.547	0.52
6	Rutin	0.321	0	0.292	0	0	0.747	0.512	0.893	0	0.893
7	Naringenin	0.213	0.237	0.476	0.578	0.462	0.709	0.868	0.822	0.642	0.557
8	Limonene	0.145	0.355	0.447	0.464	0.223	0.816	0.707	0.627	0.357	0.516
9	cis-β-ocimene	0.218	0.257	0.477	0.661	0.518	0.858	0.857	0.694	0.402	0.496
10	Allo-ocimene	0.289	0.302	0.518	0.706	0.232	0.948	0.958	0.785	0.456	0.382
11	Linalool oxide	0.43	0	0.341	0.429	0.195	0.67	0.782	0.52	0	0.713
12	Linalool	0.372	0.245	0.503	0.579	0.188	0.667	0.727	0.719	0.302	0.435
13	Methyl salicylate	0.561	0.15	0.341	0.429	0.233	0.425	0.67	0.52	0.572	0.713
14	Eucalyptol	0.314	0	0.22	0.603	0.174	0.221	0.735	0.58	0	0.296
15	Palmitate acid	0.23	0	0.246	0.769	0.641	0.342	0.907	0.74	0.642	0.355
16	Oleate acid	0.425	0.226	0.516	0.681	0.713	0.499	0.886	0.791	0.547	0.361
17	Linoleat acid	0.58	0.235	0.49	0.648	0.449	0.545	0.87	0.763	0.518	0.333
Average		0.340	0.197	0.438	0.543	0.393	0.620	0.823	0.709	0.422	0.512
Standard deviation		0.123	0.117	0.128	0.190	0.207	0.185	0.115	0.100	0.235	0.155

Score prediction ≥0.5 is marked in dark green Score prediction is between 0.3 and 0.5, it is marked in light green Score prediction ≤0.3 is marked in yellow

In Table-3, the prediction scores for the 17 metabolites’ roles in providing anthelmintic effects are categorized by three different colors. Compounds scoring above 0.5 in prediction are represented in dark green, 0.3–0.5 in light green, and below 0.3in yellow. The cholinergic and neurotransmitter antagonistic properties of this agent enhance its potential as an anthelmintic, with prediction scores of 0.301 and 0.416, respectively. Oleic acid and pyrogallol exhibited the greatest cholinergic and neurotransmitter antagonist activities, with scores of 0.713 and 0.66, respectively. In worms, this cholinergic antagonist can induce muscle paralysis and death. The two receptors identified by You *et al*. [50] are crucial for comprehending the mechanism of anthelmintic drugs through their effects on *Schistosoma haematobium*, *Schistosoma mansoni*, and *Schistosoma japonicum*. Inhibit the function of the ubiquinol-cytochrome c reductase enzyme. The QSAR prediction results show an average score of 0.807, meaning that if the Pa score is >0.7, the chance of this mechanism being experimentally proven is very high [36, 37]. In the mitochondria of eukaryotic cells, the ubiquinol-cytochrome c reductase complex functions as an electron transfer enzyme during cellular respiration. Suppressing ubiquinol-cytochrome c reductase function impairs sugar metabolism energy production.

Seventeen secondary metabolites of *V. merrillii* seeds, on average, demonstrated a greater ability to induce Ca^+2^ channels compared to praziquantel, with a score of 0.438 versus 0.125. Increased Ca^+2^ channel activity leads to endogenous Ca^+2^ release and tetanic contractions/paralysis in the worm. In the tapeworm *Hymenolepis diminuta*, praziquantel induces contraction and expulsion from the digestive tract by eliciting endogenous Ca^+2^release from cells. In schistosomes, this compound triggers muscle contractions and paralysis, as well as the creation of balloon-like structures on the surface of the tegument that serve as antigens for antibodies and are subject to phagocytosis. Seventeen secondary metabolites of *V. merrillii* seeds, on average, exhibit considerable potential as apoptotic agonists, with a Pa score of 0.620. The caspase-3 stimulant and TP53 expression enhancer mechanisms increased the potency of the apoptosis agonist, as indicated by Pa scores of 0.512 and 0.709. Thioredoxin enzyme activity inhibition is a reported mechanism for anthelmintic medications [39]. Seventeen secondary metabolites of *V. merrillii* seeds have an average Pa score of 0.543 for inhibiting thioredoxin enzyme activity (Table 3).

### Molecular docking

Seventeen *V. merrillii* compounds’ anthelmintic potential was confirmed using molecular docking with trematode worms. Based on the thioredoxin model by Shukla *et al*. [39], the thioredoxin enzyme was selected as the molecular docking target. A 3D model of *F. hepatica* worm’s thioredoxin enzyme is accessible online at https://www.rcsb.org/structure/2VIM. *Paramphistomum* spp. worms and their enzyme’s amino acid sequence have not been identified yet. *In silico* studies were exclusively conducted on *F. hepatica*.

Previously, thioredoxin ligands and enzymes were prepared with minimum system energy by adding hydrogen atoms, partial charges, and conditioning the system. Next, the “binding site” area was identified using MOE software “site finder.” Given the absence of a native ligand for the downloaded thioredoxin 2VIM receptor, the molecular docking was carried out utilizing the “blind docking” technique at the reported target “binding site” [39]. ∆Gbinding (kcal/mol) indicates the energy released upon ligand-receptor interaction, while its visualization reveals the orientation and position of the inhibitor.

The results indicate that the test ligands, including the routine compound with the strongest inhibitory effect (−11.65 kcal/mol), gallic acid (−11.07 kcal/mol), caffeic acid (−9.75 kcal/mol), and eucalyptol (−5.59 kcal/mol), all demonstrate potential as thioredoxin enzyme inhibitors. The stronger the affinity between an enzyme and its ligand, the more negative the ∆Gbinding value. The strength of this affinity is directly proportional to the value of the inhibition constant (Ki), which indicates the level of the compound’s ability to inhibit the activity of the target enzyme [51, 52].

**Figure-2:**
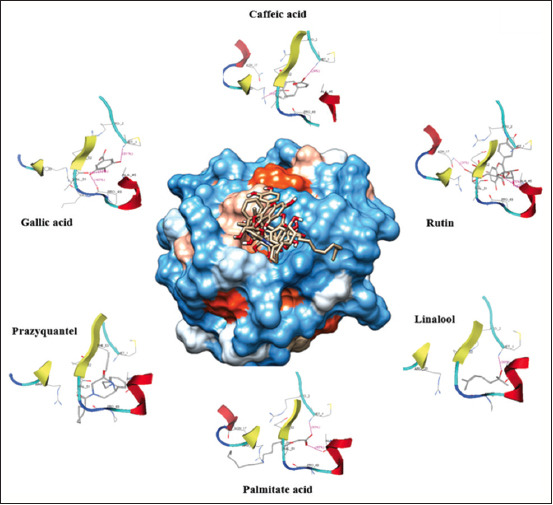
Visualization of molecular docking of five secondary metabolites of *Veitchia merrillii* with the strongest affinity compared with the praziquantel control for the *Fasciola hepatica* thioredoxin enzyme.

Seventeen *V. merrillii* compounds, based on molecular docking, exhibited strong anthelmintic potential, as evidenced by a ∆Gbinding value of −7.45 kcal/mol for praziquantel as a control. The strength of the ligand-receptor complex interaction is significantly affected by the presence of hydrogen bonds. Only those compounds with the strongest binding affinity form hydrogen bonds with thioredoxin. The docking results obtained from the complete value of ΔGbinding17 bioactive compounds are shown in Table-4 and Figure-2.

**Table-4:** ∆G*_binding_* and hydrogen bonds of 17 compounds in *V. merilli* fruit seeds

Compounds	PubChem CID	∆Gbinding(kcal/mol)	Hydrogen bond
Gallic acid	370	–11,0716	Met 1(2x), Pro 49, Val 51
Pyrogallol	1057	–9,2807	Pro 49, Val 51, Glu 52
Caffeic acid	689043	–9,7496	Met 1(2x), Arg 20
Vanillic acid	8468	–8,4007	Asn 17, Pro 49
Syringic acid	10742	–8,3156	Met 1, Ala 45
Rutin	5280805	–11,6546	Asn 17, Ala 45(2x)
Naringenin	439246	–8,8228	Val 51
Limonene	22311	–5,8445	-
cis-β-ocimene	5320250	–5,6785	-
Allo-ocimene	5368821	–5,5986	-
Linalool oxide	102611	–7,6026	Arg 2, Asn 17, Glu 52
Linalool	6549	–7,7762	Met 1(2x)
Methyl salicylate	4133	–7,6671	Val 51
Eucalyptol	2758	–5,5880	-
Palmitic acid	985	–8,8364	Met 1(2x), Ala 45
Oleic acid	445639	–7,3524	Met 1(2x)
Linoleic acid	5280450	–7,4306	Val 51
Praziquantel (Control)	370	–7,4473	-

**Figure-3:**
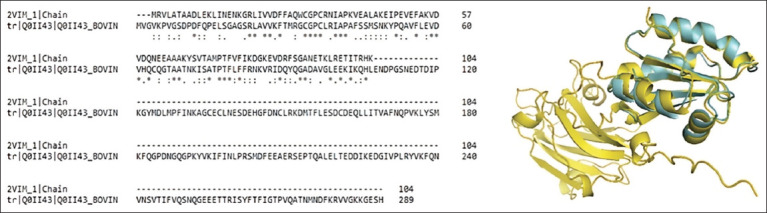
Sequence alignment and superposition of the 3D structure of *Fasciola hepatica* thioredoxin (cyan) with *Bos taurus* (yellow).

The molecular docking results demonstrate the efficacy of 17 *V. merrilli* compounds as potential anthelmintics against *F. hepatica* in cattle (*Bos taurus*). Instead of docking the *F. hepatica* worm’s molecule with the thioredoxin enzyme, alignment of their sequences and superposition of their 3D structures through the thioredoxin enzyme can suffice for comparison. Seventeen *V. merrillii* metabolites may inhibit thioredoxin enzyme in cattle as they do in *F. hepatica*. The thioredoxin enzyme’s crucial function in maintaining redox homeostasis, proliferation, and DNA synthesis [53–56] in cattle renders them susceptible to difficulties. According to Figure-3, sequence alignment and 3D structure superposition of thioredoxin enzyme from these two species reveal no similarities.

### Lipinski’s rule analysis

Lipinski’s rule applies to orally administered active compounds. Lipinski’s rule parameters, which measure the solubility and intestinal permeability of compounds in the gastrointestinal tract, serve as the foundation for estimating the oral bioavailability of active substances [57]. The test compound can meet Lipinski’s rules except for one parameter. The rules that must be met are log p ≤5, molecular weight ≤500 g/mol, hydrogen bond donor ≤5, and hydrogen bond acceptor ≤10 [58]. Table-5 presents the Lipinski parameters for 17 *V. merrillii* metabolite compounds.

**Table-5:** Lipinsks rule parameters for 17 secondary metabolite compounds of *Veitchia merrillii* fruit seeds.

Compounds	Molecular weight (<500 g/mol)	LogP (<5)	Hydrogen bond		Lipinski’s rules of five

			Donor (<5)	Acceptor (<10)	
Gallic acid	170.12	0.5	4	5	Yes
Pyrogallol	126.11	0.97	3	3	Yes
Caffeic acid	180.16	0.97	3	4	Yes
Vanillic acid	168.15	1.40	2	4	Yes
Syringic acid	198.17	1.54	2	5	Yes
Rutin	610.52	1.58	10	16	No
Naringenin	272.75	1.75	3	5	Yes
Limonene	136.23	2.72	0	0	Yes
cis-β-ocimene	136.23	2.80	0	0	Yes
Allo-ocimene	136.23	2.93	0	0	Yes
Linalool oxide	170.25	2.56	1	2	Yes
Linalool	154.25	2.70	1	1	Yes
Methyl salicylate	152.15	2.03	1	3	Yes
Eucalyptol	154.25	2.58	0	1	Yes
Palmitic acid	256.42	3.85	1	2	Yes
Oleic acid	282.46	4.27	1	2	Yes
Linoleic acid	280.45	4.14	1	2	Yes
Praziquantel (control)	312.41	3.00	22	2	Yes

### Prediction of absorption, distribution, metabolism, and toxicity

Predicting pharmacokinetic and toxic properties through the web-based pkCSM platform is crucial to prevent costly and unnecessary drug development failures. Seventeen secondary metabolites of *V. merillii* seeds’ ADME and toxicity predictions are presented in Table-6. The human intestinal absorption value and Caco-2 cell permeability were the absorption predictions evaluated. Plasma protein binding (PPB), cytochrome P450 (CYP) inhibitor metabolic profile, and toxicity (AMES and hepatotoxicity tests) were assessed. The absorption, distribution, metabolism, and toxicity data are presented in Table-6. The HIA value measures the extent of intestinal absorption of an active substance in humans. A compound is categorized as being well absorbed if the % HIA value is in the range of 70%–100%, adequate in the range of 20%–70%, and poor in the range of 0%–20% [59]. Fifteen compounds exhibit desirable HIA values, and 2 of them fall within the 20%–70% range. Eucalyptol had the highest HIA value, at 96.51%, while routine had the lowest, with only 23.45%. The *in vitro* oral absorption of active substances was predicted using Caco-2 cell modeling. In the pkCSM model, a Caco-2 cell permeability value >0.9 × 10^−6^ cm/s is indicative of high permeability. There are 11 of 17 compounds with values >0.9 × 10^−6^ cm/s, whereas there are five compounds with values <0.9 × 10^−6^ cm/s, namely, gallic acid, syringic acid, caffeic acid, vanillic acid, and routine.

**Table-6:** Prediction of absorption, distribution, metabolism, elimination, and toxicity of 17 secondary metabolites of *Veitchia merrillii* fruit seeds.

Compounds	Absorption		Distribution		Metabolism							Carcinogenic
		
	HIA (%)	Caco-2	Plasma protein binding (%)	blood brain barrier	3A4 substrate	2D6 substrate	1A2 inhibitor	C19 inhibitor	2C9 inhibitor	2D6 inhibitor	3A4 inhibitor	

		Log (10^−6^cm/s)										
Gallic aci	43.37	−0.081	38.3	−1.855	No	No	No	No	No	No	No	-
Pyrogallol	83.55	1.12	28.8	−0.441	No	No	No	No	No	No	No	-
Caffeic acid	69.41	0.634	38,7	−0.647	No	No	No	No	No	No	No	+
Vanillic acid	78.15	0.33	50.2	−0.38	No	No	No	No	No	No	No	-
Syringic acid	73.07	0.495	39.1	−0.191	No	No	No	No	No	No	No	-
Rutin	23.45	−0.949	81.3	−1.898	No	No	No	No	No	No	No	-
Naringenin	91.31	1.029	93.6	−0.578	No	No	Yes	No	No	No	No	-
Limonene	95.89	1.401	61.4	0.732	No	No	No	No	No	No	No	-
cis-β-ocimene	94.73	1.406	23.9	−1.848	No	No	No	No	No	No	No	-
Allo-ocimene	95.45	1.419	25.4	0.746	No	No	No	No	No	No	No	+
Linalool oxide	93.91	1.584	46	0.368	No	No	No	No	No	No	No	-
Linalool	93.16	1.493	51.6	0.598	No	No	No	No	No	No	No	-
Methyl salicylate	89.46	1.202	51	−0.222	No	No	No	No	No	No	No	-
Eucalyptol	96.51	1.485	44.7	0.368	No	No	No	No	No	No	No	-
Palmitic acid	92	1.558	89.9	−0.111	No	Yes	No	No	No	No	No	-
Oleic acid	91.82	1.563	94.8	−0.168	Yes	Yes	No	No	No	No	No	-
Linoleic acid	92.33	1.57	94.6	−0.142	Yes	Yes	No	No	No	No	No	-
Praziquantel	93.42	1.759	55.2	0.468	No	Yes	No	Yes	No	No	No	-

A PPB value above 90% indicates strong protein binding while below 90% indicates weak protein binding, enabling effective drug distribution [40]. According to Table-6, naringenin has a PPB value exceeding 90%. The blood–brain barrier (BBB) is another distribution parameter. A drug’s concentration in the brain is signified by its BBB value. Determining the drug’s capacity to permeate the BBB relies on this parameter. Molecules with values >0.3 in the pkCSM predictive model are assumed to readily cross the BBB, whereas those below −1 are poorly distributed in the brain. Allo-ocimene produces the highest BBB value of 0.746, allowing it to penetrate the BBB among the five identified compounds (Table-6).

A drug’s metabolic characteristics are assessed based on its ability to inhibit cytochrome enzymes. CYP isozymes, a superfamily accounting for significant drug elimination through metabolic biotransformation [60]. There are five main isoforms of CYP450, including CYP1A, CYP2C19, CYP2C9, CYP2D6, and CYP3A4 [40]. Pharmacokinetic-related drug interactions leading to toxic side effects or unwanted drug reactions are often caused by the inhibition of this specific isoenzyme [61]. The CYPenzyme’s main isoform may be influenced by naringin compounds, palmitic acid, oleic acid, and linoleic acid (Table-6). The safer induction/inhibition of cytochrome enzymes is estimated compared to the praziquantel control, which can induce/inhibit multiple types of cytochromes.

The AMES test assesses carcinogenic potential in the toxicity profile. The AMES test evaluates bacteria’s susceptibility to a compound’s mutagenic effects. A positive test indicates that the compound is mutagenic and, therefore, may act as a carcinogen [62]. Be cautious of the mutagenic or carcinogenic potential of the caffeic acid and allo-cimene compounds during therapy (Table-6).

## Conclusion

This study found that *in vitro V. merrillii* seed extract killed trematode worms in *Paramphistomum* spp. by inducing mortality and damaging their tegument. The seeds of *V. merrillii* exhibit *in silico* anthelmintic activity against *F. hepatica* similar to that of praziquantel, employing multiple mechanisms, including microtubule inhibition, Ca^+2^ channel activation, thioredoxin inhibition, caspase three stimulation, apoptosis induction, ubiquinol-cytochrome c reductase inhibition, neurotransmitter action, and cholinergic antagonism, as verified by QSAR analysis. The thioredoxin enzyme is inhibited more effectively by most compounds in *V. merrillii* seeds, as shown by molecular docking studies. According to Lipinski’s rule analysis, all compounds except for rutin are suitable for oral administration. The seeds of *V. merrillii* show promise as an anthelmintic for trematode worms from potential plant sources.

## Authors’ Contributions

FA: Conceived and designed the *in vitro* study. HV: Carried out *in vitro* tests in the parasitology laboratory. MH: Preparation of sample extracts. FF: Conceived, designed, and analyzed *in silico* study. WES: *in vitro* treatment. All authors have read, reviewed, and approved the final manuscript.

## References

[1] Cai W, Cheng C, Feng Q, Ma Y, Hua E, Jiang S, Hou Z, Liu D, Yang A, Cheng D, Xu J, Ta J (2023). Prevalence and risk factors associated with gastrointestinal parasites in goats (*Capra hircus*) and sheep (*Ovis aries*) from three provinces of China. Front. Microbiol.

[2] Brasil B.S.A.F, Nunes R.L, Bastianetto E, Drummond M.G, Carvalho D.C, Leite R.C, Molento M.B, Oliveir D.A.A (2012). Genetic diversity patterns of *Haemonchus placei* and *Haemonchus contortus* populations isolated from domestic ruminants in Brazil. Int. J. Parasitol.

[3] Soulsby E.J.L. (1982). Helminths, Arthropods and *Protozoa* of Domesticated Animals.

[4] Rizwan H.M, Usman M, Naeem M.A, Farid M.U, Younus M, Sajid M.S, Tahir U.B, Luqman N, Abbas H, Ateeq M.K, Taseer M.S.A, Asi M (2022). Prevalence of ruminant paramphistomosis and comparative histopathology of the infected rumens in Narowal District, Punjab, Pakistan. Helminthologia.

[5] Martindah E, Sawitri D.H, Wardhana AH, Ekawast F (2023). Prevalence of amphistomes and *Fasciola* in large ruminants reared by smallholders in Lampung and Banten Provinces, Indonesia. Vet. World.

[6] Meguini M.N, Righi S, Bouchekhchoukh M, Sedraoui S, Benakhl A (2021). Investigation of flukes (*Fasciola hepatica* and *Paramphistomum* spp.) parasites of cattle in north-eastern Algeria. Ann. Parasitol.

[7] Githiori J.B, Athanasiadou S, Thamsbor S.M (2006). Use of plants in novel approaches for control of gastrointestinal helminths in livestock with emphasis on small ruminants. Vet. Parasitol.

[8] Miller S.A, Ferreira J.P, LeJeun J.T (2022). Antimicrobial use and resistance in plant agriculture:A one health perspective. Agriculture.

[9] Magryś A, Olender A, Tchórzewsk D (2021). Antibacterial properties of *Allium sativum* L. against the most emerging multidrug-resistant bacteria and its synergy with antibiotics. Arch. Microbiol.

[10] Ndjonka D, Agyare C, Lüersen K, Djafsia B, Achukwi D, Nukenine E.N, Hensel A, Lieba E (2011). *In vitro* activity of Cameroonian and Ghanaian medicinal plants on parasitic (*Onchocerca ochengi*) and free-living (*Caenorhabditis elegans*) nematodes. J. Helminthol.

[11] Vidyadhar S, Saidulu M, Gopal T.K, Chamundeeswari D, Rao U, Banj D (2010). *In vitro* anthelmintic activity of the whole plant of *Enicostemma littorale* by using various extracts. Int. J. Appl. Biol. Pharm. Technol.

[12] Jeyathilakan N, Murali K, Anandaraj A, Latha B.R, Basit S.A (2010). The anthelmintic activity of essential oils of *Cymbopogan nardus* and *Azadirachta indica* on *Fasciola gigantica*. Tamilnadu J. Vet. Anim. Sci.

[13] Shalaby H.A, El-Moghaz F.M (2013). *In vitro* effect of *Nigella sativa* oil on adult *Toxocara vitulorum*. Pak. J. Biol. Sci.

[14] Pal D, Panda K (2010). Evaluation of anthelmintic activity of aerial part of *Cynodon dactylon* Pers. Anc. Sci. Life.

[15] Ajaiyeoba E.A, Onocha P.A, Olarenwaj O.T (2001). *In vitro* anthelmintic properties of *Buchholzia coriaceae* and *Gynandropsis gynandra* extracts. Pharm. Biol.

[16] Zahir A.A, Rahuman A.A, Kamaraj C, Bagavan A, Elango G, Sangaran A, Kuma B.S (2009). Laboratory determination of the efficacy of indigenous plant extracts for parasite control. Parasitol. Res.

[17] Monteiro M.V.B, Bevilaqua C.M.L, Morais S.M, Machado L.K.A, Camurça-Vasconcelos A.L.F, Campello C.C, Ribeiro W.L.C, Mesquit M.A (2011). Anthelmintic activity of *Jatropha curcas* L. seeds on *Haemonchus contortus*. Vet. Parasitol.

[18] Jeyathilakan N, Murali K, Anandaraj A, Basit S.A (2011). *In vitro* evaluation of anthelmintic property of ethno-veterinary plant extracts against the liver fluke. Fasciola gigantica. J. Parasit. Dis.

[19] Balqis U, Darmawi D, Maryam, Muslina, Hamzah A, Daud R, Hambal M, Rinidar, Harri A, Muttaqie M, Azha A, Eliawardan E (2016). Motilitas *Ascaridia galli* dewasa dalam larutan ekstrak etanol biji palem puri (*Veitchia merrillii*). Agripet.

[20] Liu P.F, Chan Y.F (2023). The controversial roles of *Areca* nut:Medicine or toxin?. Int. J. Mol. Sci.

[21] Jannah M, Machfuzh M, Sugiarto S (2021). Potential added value of *Areca* nut products in aceh. J. Teknol. Ind. Pertanian.

[22] Sudarmanto B, Akbarrizki M, Wahidah-Mubaraka W (2022). Population of *Ascaridia galli* according to its predilection after being treated with *Areca* nut infusion and its economic analysis. J. Kedokteran Hewan.

[23] Hamzah A, Hambal M, Balqis U, Darmawi D, Maryam M, Rosmaidar R, Athaillah F, Muttaqien M, Azhar A, Ismail I (2016). *In vitro* anthelmintic activity of *Veitchia merrillii* nuts against *Ascaridia galli*. Tradit. Med. J.

[24] Sari L.M. (2021). Antioxidant Activity of *Areca* Nut to Human Health:Effect on Oral Cancer Cell Lines and Immunomodulatory Activity. IntechOpen, London.

[25] Anto E.J, Lelo A, Ilyas S, Nainggola M (2022). Effect of Ethanol extract and ethyl acetate fraction of betel nut (*Areca catechu* L.) in colonic goblet cells of mice (*Mus musculus*) given orally infective egg of *Trichuris muris*. Open Access Maced. J. Med. Sci.

[26] Murwani R, Kusumanti E, Naumov E.N (2022). *Areca catechu L*. and *Anredera cordifolia (Ten) Steenis* supplementation reduces faecal parasites and improves caecal histopathology in laying hens. Int. J. Vet. Sci. Med.

[27] Dewi P, Indriyanti D.R, Herlina L, Gunawan A.S, Lusian C.E (2022). Potency of *Areca catechu* flesh extract in inhibiting soft rot fungi of melons and bananas. J. Teknol.

[28] Asrianto A, Asrori A, Sahli I.T, Hartati R, Kurniawan F.B, Purwat R (2021). Bioaktivitas ekstrak etanol biji pinang (*Arecha catechu* L.) terhadap *Staphylococcus aureus* dan *Escherichia coli*:Bioactivity of betel nut (*Arecha catechu* L.) ethanol extract against *Staphylococcus aureus* and *Escherichia coli*. J. Sains Kesehatan.

[29] Yayuk A, Burhanuddin B, Hakim A, Loka I.N, Muti'a M (2021). Chemical content in the Sembeq traditional rituals of the Lombok Community. J. Pijar Mipa.

[30] Latifah S, Valentino N, Sari D, Sar B.S.A (2021). Species composition, and diversity of Mataram University green open space, West Nusa Tenggara. IOP Conf. Ser. Earth Environ. Sci.

[31] Chen D.J, Landis J.B, Wang H.X, Sun Q.H, Wang Q, Wan H.F (2022). Plastome structure, phylogenomic analyses and molecular dating of *Arecaceae*. Front. Plant Sci.

[32] Rodríguez-Leyes E.A, Vicente-Murillo R, González-Canavaciolo V.L, Sierra-Pérez R.C, Marrero-Delange D, Leiva-Sánche Á.T (2013). Content of grass acids in the lipid fraction of Arecaceae three-fuirt crops in Cuba [Contenido de ácidos grasos en la fracción lipídica de frutos de tres Arecaceae cultivadas en Cuba]. Rev. Cenic Cien. QuíMicas.

[33] Iyasele J.U, Uadia J.O, Akhigbe I.U, Jacob J.N, Ogbeid O.K (2022). Physico-chemical properties, chemical composition and antimicrobial activity of *Adonidia merrillii* kernel seed oil. Trop. J. Nat. Prod. Res.

[34] Vafaei A (2013). Antioxidant and Cytotoxicity Activities of *Veitchia merrillii* Fruits. The 4^th^ World Congress on Biotechnology, 23-25 Raleigh, North Carolina, USA.

[35] Jiraungkoorskul W, Sahaphong S, Tansatit T, Kangwanrangsan N, Pipatshukia S (2005). *Eurytrema pancreaticum*:The *in vitro* effect of praziquantel and triclabendazole on the adult fluke. Exp. Parasitol.

[36] Anzali S, Barnickel G, Cezanne B, Krug M, Filimonov D, Poroiko V (2001). Discriminating between drugs and nondrugs by prediction of activity spectra for substances (PASS). J. Med. Chem.

[37] Ramadhan D.S.F, Fakih T.M, Arfa A (2020). Activity prediction of bioactive compounds contained in *Etlingera elatior* against the SARS-CoV-2 main protease:An *in silico* approach. Borneo J. Pharm.

[38] Filimonov D.A, Lagunin A.A, Gloriozova T.A, Rudik A.V, Druzhilovskii D.S, Pogodin P.V, Poroiko V.V (2014). Prediction of the biological activity spectra of organic compounds using the PASS online web resource. Chem. Heterocycl. Comp.

[39] Shukla R, Shukla H, Kalita P, Sonkar A, Pandey T, Singh D.B, Kumar A, Tripath T (2018). Identification of potential inhibitors of *Fasciola gigantica* thioredoxin1:Computational screening, molecular dynamics simulation, and binding free energy studies. J. Biomol. Struct. Dyn.

[40] Pires D.E, Blundell T.L, Asche D.B (2015). pkCSM:Predicting small-molecule pharmacokinetic and toxicity properties using graph-based signatures. J. Med. Chem.

[41] Chafton L.A. (2006). The Effect of a Condensed Tannin-Containing Forage, *Sericea lespedeza*, on Existing and Challenging Infections of *Haemonchus contortus* in Sheep. Thesis. The Graduate Faculty of the Louisiana State University and Agricultural and Mechanical College.

[42] Bachaya H.A, Iqbal Z, Khan M.N, Shindu Z.D, Jabba A (2009). Anthelmintic activity of *Ziziphus nummularia* (bark) and *Acacia nilotica* (fruit) against *Trichostrongylid nematodes* of sheep. J. Ethnopharmacol.

[43] Mali R.G, Meht A.A (2008). A review on anthelmintics plants. Nat. Prod. Radiance.

[44] Lateef M, Iqbal Z, Khan M.N, Akhtar M.S, Jabba A (2003). Anthelmintic activity of *Adhatoda vesica* roots. Int. J. Agric. Biol.

[45] Lakshmi V, Joseph S.K, Srivastava S, Verma S.K, Sahoo M.K, Dube V, Mishra S.K, Murth P.K (2010). Antifilarial activity *in vitro* and *in vivo* of some flavonoids tested against *Brugia malayi*. Acta Trop.

[46] Hoet S, Pieters L, Muccioli G.G, Habib-Jiwan J.L, Opperdoes F.R, Quetin-Leclerc J (2007). Antitrypanosomal activity of triterpenoids and sterols from the leaves of *Strychnos spinosa* and related compounds. J. Nat. Prod.

[47] Siddiqui B.S, Afshan F, Ghiasuddin G, Faizi S, Naqvi S.N.H, Tari R.M (2000). Two insecticidal tetranortriterpenoids from *Azadirachta indica*. Phytochemistry.

[48] Mansour T.E, Mansou J.M (2002). Target Kemoterapi pada Parasit. Cambridge University Press, UK.

[49] Smyth J.D, Halto D.W (1983). Fisiologi Trematoda. Cambridge University Press, Inggris, UK.

[50] You H, Liu C, Du X, McManu D.P (2017). Acetylcholinesterase and nicotinic acetylcholine receptors in schistosomes and other parasitic helminths. Molecules.

[51] Trott O, Olso A.J (2010). AutoDock vina:Improving the speed and accuracy of docking with a new scoring function, efficient optimization, and multithreading. J. Comput. Chem.

[52] Shoichet B.K, McGovern S.L, Wei B, Irwi J.J (2002). Lead discovery using molecular docking. Curr. Opin. Chem. Biol.

[53] Oberacker T, Kraft L, Schanz M, Latus J, Schricke S (2023). The importance of thioredoxin-1 in health and disease. Antioxidants (*Basel*).

[54] Muri J, Heer S, Matsushita M, Pohlmeier L, Tortola L, Fuhrer T, Conrad M, Zamboni N, Kisielow J, Kop M (2018). The thioredoxin-1 system is essential for fueling DNA synthesis during T-cell metabolic reprogramming and proliferation. Nat. Commun.

[55] Hasan AA, Kalinina E, Tatarskiy V, Shti A (2022). The thioredoxin system of mammalian cells and its modulators. Biomedicines.

[56] Kansal H, Chopra V, Garg K, Sharm S (2023). Role of thioredoxin in chronic obstructive pulmonary disease (COPD):A promising future target. Respir. Res.

[57] Ramachandran B, Kesavan S, Rajkuma T (2016). Molecular modeling and docking of small molecule inhibitors against NEK2. Bioinformation.

[58] Lipinski C.A. (2004). Lead- and drug-like compounds:The rule-of-five revolution. Drug Discov. Today. Technol.

[59] Cheng J, Palva A.M, de Vos W.M, Satokar R (2013). Contribution of the intestinal microbiota to human health:From birth to 100 years of age. Curr. Top. Microbiol. Immunol.

[60] Bibi Z (2008). Role of cytochrome P450 in drug interactions. Nutr. Metab. (*Lond*).

[61] Kirchmair J, Göller A.H, Lang D, Kunze J, Testa B, Wilson I.D, Glen R.C, Schneide G (2015). Predicting drug metabolism:Experiment and/or computation?. Nat. Rev. Drug Discov.

[62] Mortelmans K, Zeige E (2000). The Ames *Salmonella*/microsome mutagenicity assay. Mutat. Res.

